# RNA interference identifies domesticated viral genes involved in assembly and trafficking of virus-derived particles in ichneumonid wasps

**DOI:** 10.1371/journal.ppat.1008210

**Published:** 2019-12-13

**Authors:** Ange Lorenzi, Marc Ravallec, Magali Eychenne, Véronique Jouan, Stéphanie Robin, Isabelle Darboux, Fabrice Legeai, Anne-Sophie Gosselin-Grenet, Mathieu Sicard, Don Stoltz, Anne-Nathalie Volkoff

**Affiliations:** 1 DGIMI, INRA, University of Montpellier, Montpellier, France; 2 UMR 1349 INRA/Agrocampus Ouest/Université Rennes 1, Institut de Génétique, Environnement et Protection des Plantes (IGEPP), Le Rheu, France; 3 Université Rennes 1, INRIA, CNRS, IRISA, Rennes, France; 4 ISEM, University of Montpellier, CNRS, IRD, EPHE, Montpellier, France; 5 Department of Microbiology and Immunology, Dalhousie University, Halifax, Canada; University of Georgia, UNITED STATES

## Abstract

There are many documented examples of viral genes retained in the genomes of multicellular organisms that may in some cases bring new beneficial functions to the receivers. The ability of certain ichneumonid parasitic wasps to produce virus-derived particles, the so-called ichnoviruses (IVs), not only results from the capture and domestication of single viral genes but of almost entire ancestral virus genome(s). Indeed, following integration into wasp chromosomal DNA, the putative and still undetermined IV ancestor(s) evolved into encoding a ‘virulence gene delivery vehicle’ that is now required for successful infestation of wasp hosts. Several putative viral genes, which are clustered in distinct regions of wasp genomes referred to as IVSPERs (Ichnovirus Structural Protein Encoding Regions), have been assumed to be involved in virus-derived particles morphogenesis, but this question has not been previously functionally addressed. In the present study, we have successfully combined RNA interference and transmission electron microscopy to specifically identify IVSPER genes that are responsible for the morphogenesis and trafficking of the virus-derived particles in ovarian cells of the ichneumonid wasp *Hyposoter didymator*. We suggest that ancestral viral genes retained within the genomes of certain ichneumonid parasitoids possess conserved functions which were domesticated for the purpose of assembling viral vectors for the delivery of virulence genes to parasitized host animals.

## Author summary

Thousands of parasitic wasp from the ichneumonid family rely on virus-derived particles, named Ichnoviruses (Polydnavirus family), to ensure their successful development. The particles are produced in a specialized ovarian tissue of the female wasp named calyx. Virions are assembled in the calyx cell nuclei and stored in the oviduct before being transferred to the parasitoid host upon female wasp oviposition. Genes encoding proteins associated with the particles had been previously identified. These genes are localized in clusters of genes in the wasp genome (named IVSPER for “Ichnovirus structural proteins encoding regions”), they are specifically transcribed in the calyx but not encapsidated. IVSPER genes were thus hypothesized to derive from the integration of a virus, however still undetermined. Indeed, none of the identified genes had similarity to known sequence, making in addition unclear their function in particle production. In this work, we use the RNA interference technology to decipher the function of six IVSPER genes from the ichneumonid wasp *Hyposoter didymator*. Thanks to this approach, combined with transmission electron microscopy, we show that the studied IVSPER genes are required in different steps of particle morphogenesis and trafficking, and that their functions are those expected of a typical virus.

## Introduction

Integration of viral sequences is widespread in eukaryotic DNA and is documented as a major source of genome diversification [1]. In animal species, endogenous viral elements (EVEs) most commonly derive from the integration of retroviral cDNAs into germ-line tissue; indeed, approximately 8% of the human genome consists of endogenous retroviruses sequences [2,3]. More recently, the large scale acquisition of genomic data for many multicellular organisms has revealed the presence of sequences derived not only from retroviruses but also from a wide range of both DNA and RNA viruses [1,4–9]. While most viral gene functions are lost over time due to accumulation of random mutations, sometimes a captured viral gene is maintained in the host genome and supplies a valuable function to the latter, or is exapted by the host to serve a new function. For example, the syncytins, a family of mammalian genes required for successful placentation, derive from genes encoding retrovirus envelope proteins, now conserved for other purposes [10]. Similarly, the neuronal protein Arc, which assembles into capsid-like structures that mediate communication between mammalian nerve cells, derives from the retroviral polyprotein Gag [11,12]. Exaptation of genes from non-retroviruses is also documented. For instance, a non-structural densovirus protein promotes the dispersal of pea aphids by inducing the production of winged offspring, allowing them to escape crowded conditions [13]. Such exaptations, however, represent domestication of single viral genes, rather than almost complete genomes. To our knowledge, among eukaryotic organisms, only the polydnaviruses (PDVs) provide examples of the latter.

The *Polydnaviridae* family, as currently defined, is comprised of two unrelated taxa which share polydisperse packaged genomes and a common life cycle reviewed in [14]. PDVs are associated with certain lineages of parasitic wasps, or parasitoids, belonging to the families Braconidae and Ichneumonidae. Virus-like particles, produced exclusively in female wasp ovaries, are released into the oviducts, from which they are injected into host larvae (usually lepidopteran caterpillars) during oviposition. In these biological systems, PDVs are necessary for successful parasitoid development within their hosts.

PDVs have been referred to as ‘viruses’ because they produce particles enclosing genetic material, that resemble those of known viruses [15,16]. However, whether or not the particles described in parasitoids result from EVEs has long puzzled the scientific community. It is known today that two categories of DNA sequences involved in the PDV life cycle are carried within wasp genomes. Only the first, which carry a battery of so-called “virulence genes” of predicted insect origin, are amplified, circularized, and then packaged to be transferred to the parasitoid’s host; expression of these genes in the parasitized host is required for successful parasitism. The second category of PDV sequences present in the wasp genome encodes the genes putatively required for virion production, which are not encapsidated. Accordingly, PDV virions are non-replicative, making them different from “true” viruses. Nonetheless, the genes needed to produce the particles clearly have viral ancestral origins [17,18]. Astonishingly, the two PDV taxa result from completely unrelated viruses that integrated into the genome of two independent wasp lineages and were domesticated to perform similar functions, apparently by convergent evolution [18]. One of the PDV taxa, the bracoviruses (BVs), originated following acquisition of a complete nudivirus genome by an ancestral microgastrine wasp [17]. The genes involved in BV virion production are thus related to nudiviral genes, knowledge that facilitated the assignment of their functional roles [19]. Conversely, the origin of the other PDV taxon, the ichnoviruses (IVs), while certainly viral [18,20], is presently unknown.

The broad context of virus-derived particle production in icheumonid wasps, albeit poorly understood, has been described in several species [21–23]. Briefly, these events are restricted to the ovarian calyx, and begin during the pupal stage of wasp metamorphosis. Immature particles, here referred to as “subvirions”, consist of a lenticular nucleocapsid surrounded by a unit membrane envelope formed within calyx cell nuclei. Nucleocapsid and membrane co-assemble at the periphery of relatively homogeneous electron dense regions termed “virogenic stroma”. Then, by a process unique to the IV taxon, enveloped subvirions bud through the nuclear envelope, subsequently losing both nuclear membranes upon transit through the cytoplasm. They finally gain access to the oviduct by budding through the apical plasma membrane. Consequently, mature virions released into the lumen of the oviduct consist of a nucleocapsid surrounded by 2-unit membrane envelopes, the inner corresponding to the envelope apparently assembled *de novo* in the nucleus and the outer deriving from the plasma membrane. Mature virions are then exported into the hemocoel of parasitized host larvae upon oviposition by the female wasp. Noteworthy, trafficking of virus-derived particles in icheumonid wasps is different from other nuclear replicating viruses in two respects: 1) while other viruses, such as herpesviruses, bud through the inner nuclear membrane, IVs bud through the nuclear envelope, acquiring transiently both inner and outer nuclear membranes, and 2) unlike other nuclear viruses, IV exit remains non-lytic throughout the entire lifetime of the wasps which carry them.

In the genome of icheumonid wasps producing virus-derived particles, the genes putatively derived from the viral ancestor are located in genomic regions named IVSPERs (for IchnoVirus Structural Protein Encoding Regions; [18]). All IVSPER genes are expressed specifically in the calyx of female wasps, and a number encode proteins that were detected by mass spectrometry analysis of purified IV particles [18,20]. However, because they have no similarities in databases, the functions of these genes have remained unknown. Given that particles are produced exclusively in wasp calyx cells and cannot replicate, their morphogenesis cannot be studied in the usual manner, *i*.*e*. by injecting virions into a permissive host or infecting appropriate cell lines. Accordingly, viral particle production can only be examined *in situ*, by studying what occurs in the parasitoid ovary. Here, we used an RNA interference-based approach for studying IVSPER genes function in the ichneumonid wasp *Hyposoter didymator*. Our data allows us to associate specific IVSPER genes with subvirion assembly and different steps of intracellular virion trafficking events inside calyx cells.

## Results

### Selection of IVSPER genes based on their expression pattern during wasp pupal development

**Fig 1:**
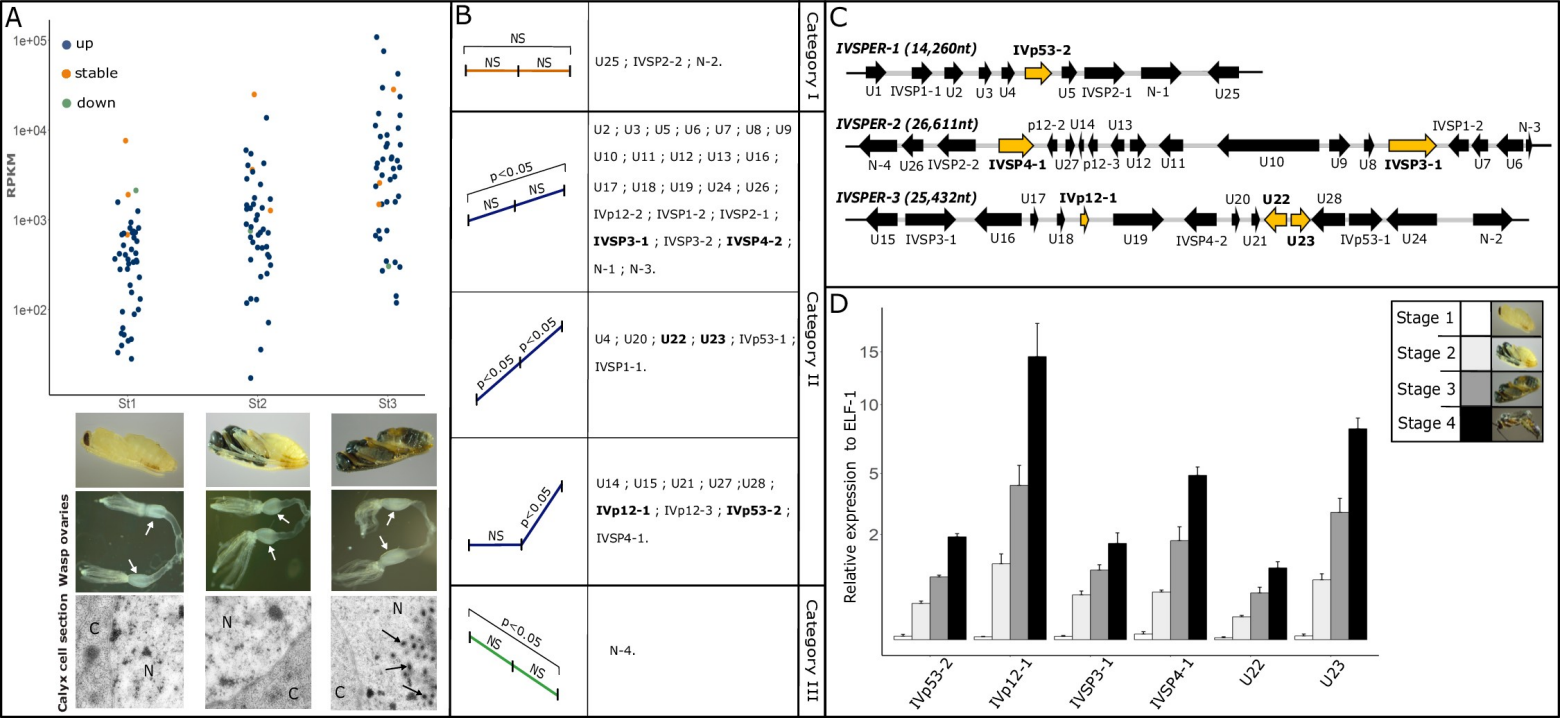
Expression pattern of IVSPER genes in *Hyposoter didymator* calyx cells during pupal developmental stages. (A) Mean reads per kilo base per million mapped reads (RPKM) values are given from RNA-seq analyses (transformed by log10). Genes with a similar expression pattern are represented by the same color (genes with stable transcript levels over pupal development time in orange, genes with increasing transcript levels in blue and those with decreased transcript levels in green). See S1 Table for raw values. The three analyzed pupal stages are shown underneath the graph: St1: pupal stage 1; St2: pupal stage 2; St3: pupal stage 3. For each stage, are shown (i) a picture of dissected wasp ovaries; white arrows indicate the calyx where virus particles are produced and (ii) TEM micrographs of calyx cells showing that the first particles are visible at stage 3. N, nucleus; C, cytoplasm; black arrows indicate viral particles. (B) Schematic representation of the different categories of expression profiles observed for IVSPER genes and list of genes in each category (see S4 Table for detail of statistical analyses). NS, no statistical differences of the RPKM values between the 2 compared pupal stages; if p<0.05, the RPKM values between the 2 compared pupal stages were considered as statistically different. (C) Schematic representation of the 3 IVSPERs identified in *H*. *didymator* genome. Arrows represent the IVSPER genes, in yellow are indicated the 6 genes selected for functional analyses. (D) Expression of the 6 selected IVSPER genes during the time course of *H*. *didymator* pupal development. Expression is given relative to the reference gene ELF-1 and at 4 pupal stages (see S1 Table for raw values and statistical analyses).

In *H*. *didymator*, subvirions and virions first become visible by transmission electron microscopy (TEM) in calyx epithelial cells at stage 3 of the wasp pupal development (Fig 1A). Nonetheless *H*. *didymator* IVSPER genes were already transcribed at pupal stage 1 as revealed by the analysis of the wasp ovarial transcriptome (Fig 1A, S1 Table). All IVSPER genes were expressed from pupal stage 1 through stage 3, although the intensity (i.e. RPKM value) of gene expression varied (Fig 1A, S1 Table). The global RNA-seq analysis also indicated differences in expression patterns: some IVSPER genes displayed transcription levels that increased from pupal stage 1 to pupal stage 3; others remained stable and one decreased over time (Fig 1B, S1 Table). To select genes potentially involved in IV particle production, we assumed that structural genes would be those displaying a temporal correlation between expression pattern and particle production: e.g., those for which transcription level increased from pupal stage 1 to pupal stage 3 (when the first particles are observed). Therefore, six IVSPER genes meeting this criterion—and encoding products previously found to be associated with the mature *H*. *didymator* virions by proteomics analyses [18]—were selected for functional analyses: *U22*, *U23*, *IVSP3-1*, *IVSP4-1*, *IVp12-1*, and *IVp53-2* (Fig 1C). Their time-course expression pattern was further analyzed using quantitative PCR (RT-qPCR) at the same time points as above, plus an additional late stage (stage 4, close to adult emergence). All six genes showed increased transcription levels over pupal development (Fig 1D, S2 Table) confirming the transcription pattern observed previously.

### dsIVp12-1 injection impairs formation of virogenic stroma

For controls, wasps were injected with non-specific dsRNA homologous to the Green Fluorescent Protein (GFP) gene. TEM observations confirmed that *H*. *didymator* Ichnovirus (HdIV) virion production was similar in dsGFP injected wasps compared to untreated wasps (see below). For the six tested IVSPER genes, injection of gene-specific dsRNA in females at pupal stage 1 resulted in a decrease of corresponding RNA levels in adult wasp abdomens (S1 Fig, S3 and S4 Tables). In addition, for each gene, we found that dsRNA injection did not affect the expression of a set of other IVSPER genes (S1 Fig, S3 and S4 Tables).

**Fig 2:**
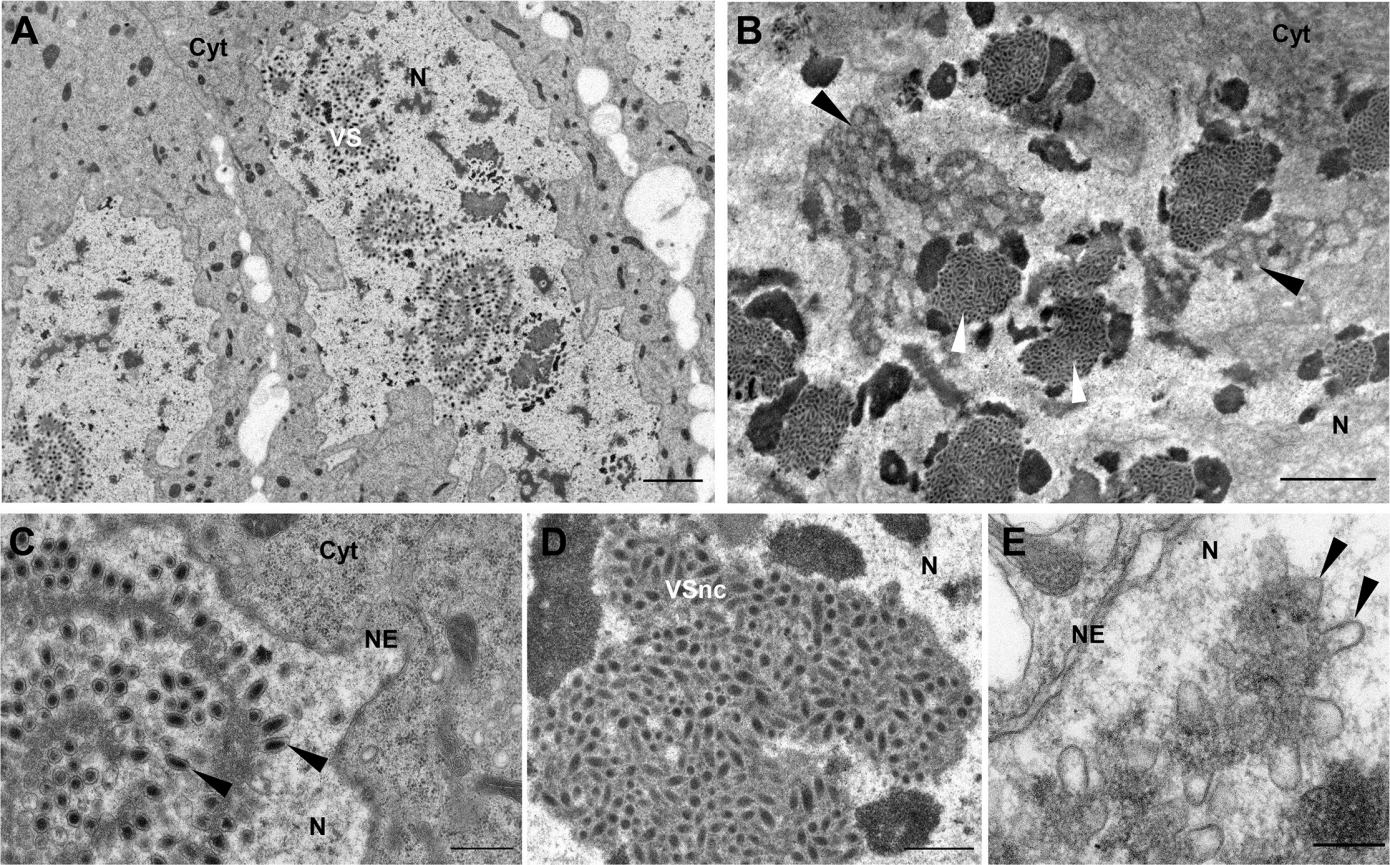
Knockdown of *IVp12-1* impairs virogenic stroma formation and subvirion assembly. (A) General view of calyx epithelial cells from a dsGFP injected (control) wasp showing the intranuclear virogenic stroma (VS) where subvirions assemble. (B) General view of a calyx cell nucleus from a dsIVp12-1 injected (treated) wasp in which two types of electron dense regions can be distinguished, one associated with membranes (black arrowheads), and another corresponding to large inclusions of nucleocapsids (white arrowheads). (C) Detail showing the virogenic stroma in control wasps (dsGFP injected) at the periphery of which enveloped nucleocapsids (i.e. subvirions) assemble (arrowheads). (D) Detail of an accumulation of non-enveloped nucleocapsids embedded in electron dense material in a dsIVp12-1 injected wasp. (E) Detail of the region associated with membranes (arrowheads) in a dsIVp12-1 injected wasp. Cyt., cytoplasm; N, nucleus; NE, nuclear envelope. Scale bars: A, B: 2 μm; C, D: 500 nm; E: 200 nm.

**Fig 3:**
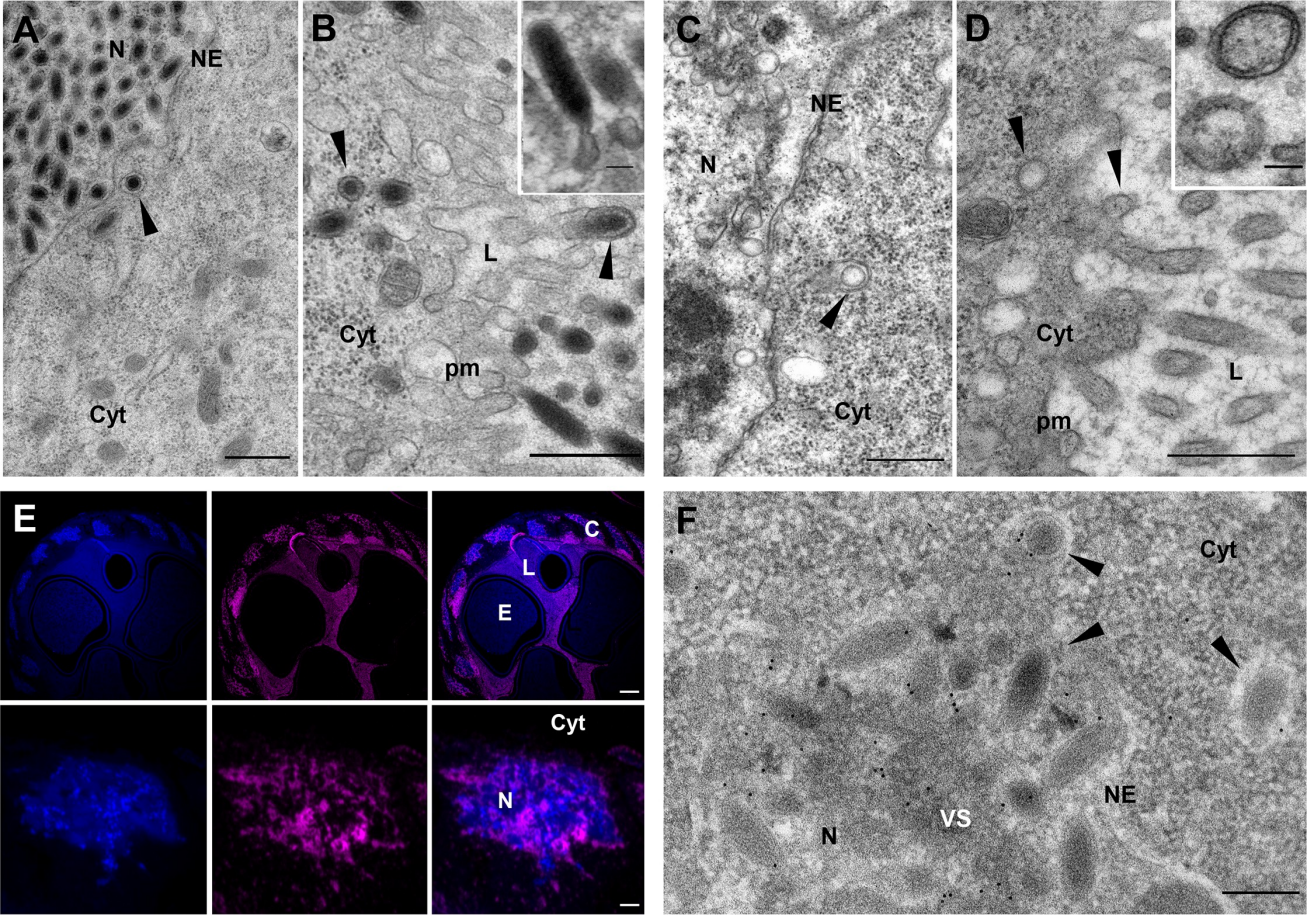
Knockdown of *IVp12-1* does not impair intracellular trafficking of the envelopes formed in the nucleus. (A) View of a calyx cell from a dsGFP injected female showing subvirions in the nucleus (N) then reaching the cytoplasm (Cyt) after budding. Arrowhead indicates a subvirion after egression from the nucleus. (B) View from a dsGFP injected female showing subvirions nearby the calyx cell plasma membrane (pm) and budding towards the calyx lumen (L). Inset shows a mature virion in the lumen, surrounded by the two envelopes. (C) View of a calyx cell from a dsIVp12-1 injected female showing vesicular envelopes formed in the nucleus which exit towards the cytoplasm by budding at the nuclear envelope (NE). Arrowhead indicates a vesicle shortly after egression from the nucleus. (D) View of a calyx cell from a dsIVp12-1 injected female showing vesicular envelopes in the cytoplasm (arrowhead) which bud at the calyx cell plasma membrane (pm) and accumulate in the oviduct lumen. Inset shows mature “defective particles”, lacking nucleocapsids, but surrounded by 2 envelopes similarly to mature virions in control wasps. (E) Immuno-labeling of a calyx section from an untreated *H*. *didymator* female using an antibody against IVp12-1. DNA labelled in blue with DAPI. IVp12-1 (labeled in red) is detected in the calyx cells nuclei and within the calyx lumen (above panel); and seems to be more intensively associated with specific nuclear spots, probably virogenic stroma (lower panel). (F) Immunogold labeling showing association of IVp12-1 with virogenic stroma (VS) and subvirions (arrowheads). C, calyx cell; Cyt., cytoplasm; L, calyx lumen; E, parasitoid egg; mv, microvilli. Scale bars: A, B, C, D: 500 nm; insets (in B, D): 100 nm; E: upper panel 20 μm, lower panel 2 μm; F: 200 nm.

The first IVSPER gene analyzed, *IVp12-1*, encodes a small 78 amino acid protein and displays a high increase in transcription levels during wasp pupation, reaching one of the highest levels of transcription observed at pupal stage 3 compared to the remaining IVSPER genes (S1 Table). After knockdown of *IVp12-1* expression, calyx cell nuclei contained no enveloped nucleocapsids, as normally observed close to the virogenic stroma in controls (Fig 2A). Instead, nucleocapsids and envelopes were each formed in distinct regions (Fig 2B, 2D and 2E). Nucleocapsids remained embedded in an amorphous material (Fig 2D) in contrast to controls (dsGFP injected or untreated wasps), where they were almost exclusively located at the periphery of the virogenic stroma (Fig 2C). On the other hand, the vesicular envelopes, lacking nucleocapsids, were able to exit the nucleus and reach the calyx lumen similarly to subvirions in control wasps (compare Fig 3A and 3B, dsGFP, and Fig 3C and 3D, dsIVp12-1). Altogether, these observations indicate that IVp12-1 is involved in correct association of membranes and nucleocapsid components at virogenic stroma and thus in subvirion assembly in specific nuclear regions. Immunolocalization assays using a IVp12-1 specific antibody and calyces from untreated *H*. *didymator* adult females show localization of the protein in the nuclei and the oviduct lumen (Fig 3E). Furthermore, a clear association of IVp12-1 with the virogenic stroma and the enveloped nucleocapsid was revealed thanks to immunogold labeling of calyces from stage 3 pupae (Fig 3F).

### dsIVSP4-1 or dsU23 injection impairs nucleocapsid assembly

**Fig 4:**
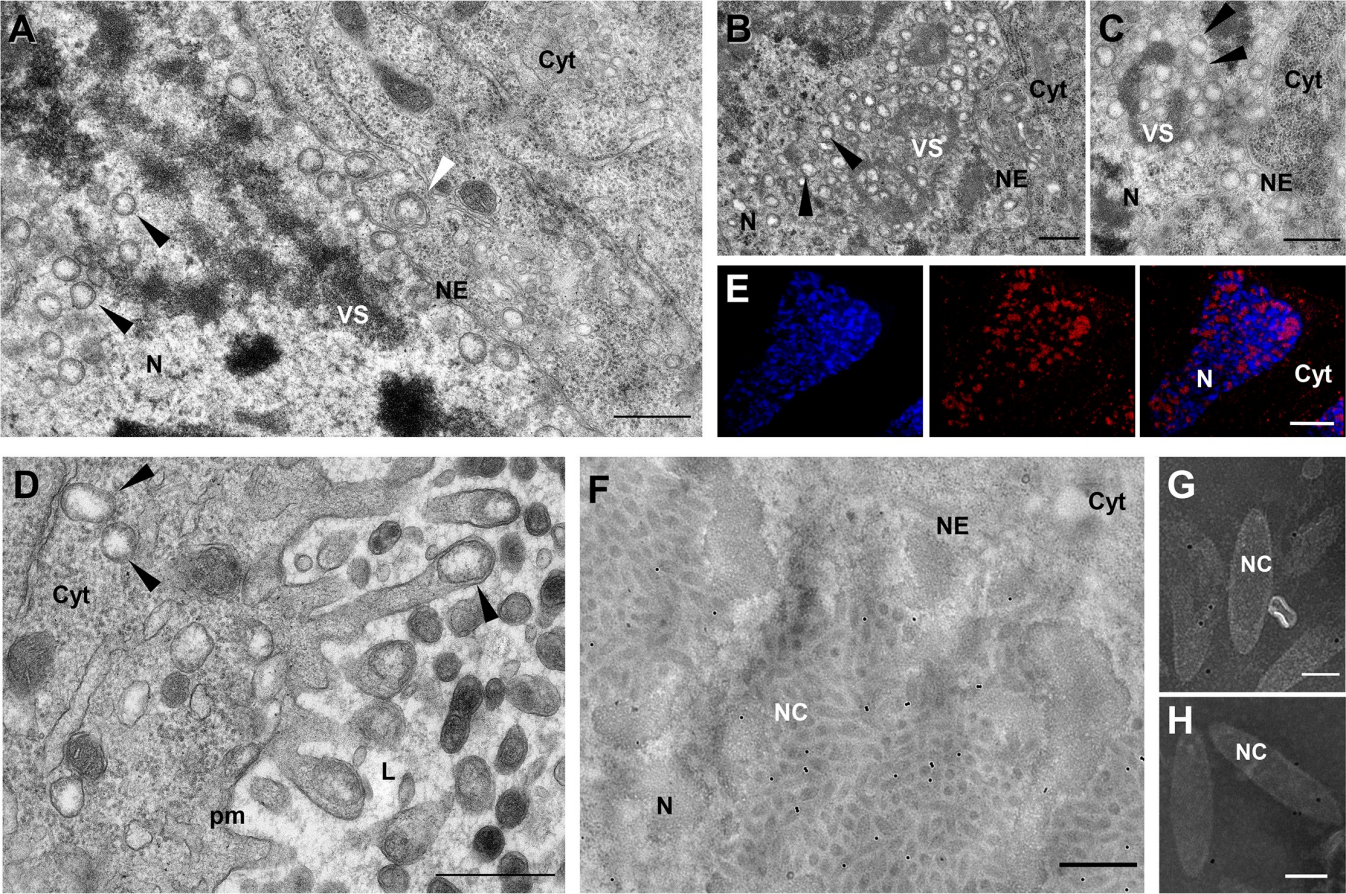
Knockdown of *U23* impairs nucleocapsid assembly. (A) Micrograph showing the virogenic stroma (VS) inside a calyx cell nucleus after treatment with dsU23: Following knockdown of U23, no nucleocapsid can be observed in association with the VS, but numerous membrane vesicles (arrowheads) are formed. The latter exit the nucleus by budding at the nuclear envelope (NE). The same step is illustrated for control dsGFP in Fig 3A. The white arrowhead indicates a vesicle, or “defective particle”, just after budding surrounded by the two nuclear membranes. (B) General view of the nucleus of a calyx cell from a dsU23 injected wasp showing absence of nucleocapsids and the virogenic stroma (VS) surrounded by membranous vesicles (arrowheads). (C) Similarly, only vesicles (arrowheads) are observed in the nucleus of a calyx cell from a dsIVSP4-1 injected wasp. (D) View of a dsU23 injected calyx cell showing defective particles (arrowheads) in the cytoplasm; they bud at the calyx cell plasma membrane (PM) and accumulate in the oviduct lumen (L). (E) Confocal laser scanning image of calyx cells from dsIVp12-1 injected females after immunolabeling using an anti-U23 antibody (red labeling). The view corresponds to a single calyx cell, characterized by an hypertrophied nucleus visualized thanks to DAPI staining of the DNA (blue labeling). In dsIVp12-1 samples, nucleocapsids and envelopes are separated in the nuclei, and the nucleocapsids form dense aggregates. Immunolabeling shows that U23 is preferentially localized in the calyx cell nucleus; patches of bright red spots are seen all over the nucleus which could correspond to the nucleocapsid aggregates. (F) Immunogold labeling of dsIVp12-1 treated calyx confirms association of U23 with the nucleocapsid (NC) aggregates. (G, H) Immunogold labeling of nucleocapsids purified from untreated females. The particles shown have lost their envelopes during sample preparation and naked nucleocapsids are labeled, confirming association of U23 with the nucleocapsid. Cyt., cytoplasm; N, nucleus. Scale bars: A, B, C, D, F: 500 nm; E: 10 μm; G, H: 100 nm.

*IVSP4-1* and *U23* code for unrelated proteins of 446 and 415 amino acids, respectively. In wasps injected with dsIVSP4-1 or dsU23, calyx cells nuclei contained no distinguishable subvirions but abundant membranes, either positioned adjacent to the virogenic stroma, or vesiculated and free in the nucleoplasm (Fig 4A, 4B and 4C). The membranes formed “defective particles” since, although lacking nucleocapsids, they exit the nucleus (Fig 4A), traffic across the cytoplasm and bud into the oviduct lumen (Fig 4D), as would normal subvirions.

The defective particles appeared to contain small amounts of material situated on the inner side of their inner envelope. To check for the presence or absence of DNA, particles were collected from wasps injected with dsGFP, dsIVSP4-1 or dsU23 respectively and used in PCR reactions with primers specific to *GlyPro2* (GenBank: AIK25735), a gene encoded by a packaged *H*. *didymator* IV DNA sequence. No amplification was observed in the dsU23 and dsIVSP4-1 samples; conversely, appropriate PCR products were amplified from control particles (S2A Fig). Absence of packaged DNA in purified “defective particles” was also confirmed by the lack of DAPI labeling in the calyx lumen of treated females compared to controls where calyx fluid was intensively stained (S2B Fig). The circular DNA molecules normally packaged into virions result from the amplification and circularization of chromosomal sequences. Absence of DNA within “defective particles” could be caused by the absence of circularized viral molecules. Assessment of the presence or absence of these circular IV DNA molecules in the nuclei of calyx cells of dsU23 or dsIVSP4-1 treated wasps was performed by PCR using ovarian genomic DNA as template and primers amplifying specifically the circular form of the Hd29 molecule (GenBank: KJ586303.1; see S2C Fig for primers position). Amplification of PCR products in all dsGFP, dsIVSP4-1 or dsU23 wasp samples indicate that circularization of IV chromosomal sequences was not eliminated by knockdown of *U23* or *IVSP4-1* (S2C Fig). Taken together, our results suggest that U23 and IVSP4-1 gene products are not involved in the production of circularized IV molecules but are necessary for nucleocapsid assembly and/or DNA packaging.

Protein localization was studied more specifically for U23. For that, we took advantage of the fact that dsIVp12-1 inhibition provides samples where nucleocapsids and envelopes are separated in the nuclei. U23 labeling of dsIVp12-1 injected calyces resulted in highly fluorescent spots within the nuclei (Fig 4E), which corresponded to the regions where nucleocapsids accumulate as confirmed by immunogold labeling (Fig 4F). Association of U23 with nucleocapsids was also confirmed by immunogold labeling of nucleocapsids released from purified control HdIV virions (Fig 4G and 4H).

### dsIVSP3-1 or dsU22 injection impairs subvirion egression from the nucleus

**Fig 5:**
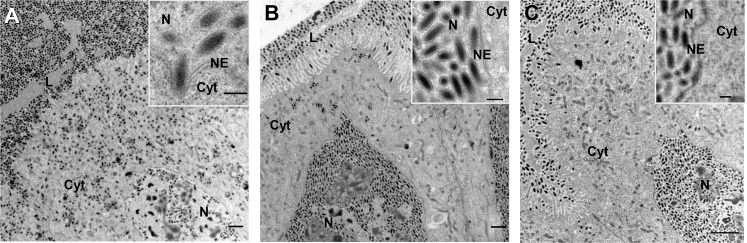
Knockdown of genes involved in virion nuclear exit. (A). Calyx section of a dsGFP injected *H*. *didymator* female showing subvirions in the nucleus (N), the cytoplasm (Cyt) and the oviduct lumen (L). Inset shows a detail of a subvirion budding at the nuclear envelope (NE). (B) Calyx section of a dsIVSP3-1 injected female. Note the large amount of subvirions in the nucleus and the few subvirions in the cytoplasm. Inset showing a detail of subvirions accumulated in the nucleus. (C) Calyx section of a dsU22 injected female. Subvirions accumulate in the nucleus and only rarely are observed budding at the nuclear envelope (Inset). Scale bars: A, B, C: 2 μm; insets: 200 nm.

*IVSP3-1* and *U22* code for unrelated proteins 630 and 288 amino acids long, respectively. Both proteins have predicted transmembrane domains in either the C-terminal (IVSP3-1) or N-terminal region (U22). Silencing of either of the 2 genes did not impair subvirion formation but led to a great accumulation of subvirions inside the calyx cell nuclei compared to ds*GFP* calyx cells (Fig 5). Budding from the nucleus to the cytoplasm seems inhibited but not totally impaired by dsRNA treatment, as virions were observed in the cytoplasm and the calyx lumen (Fig 5). IVSP3-1 and U22 therefore appear to be two proteins necessary for efficient budding of nucleocapsids out of the calyx cell nucleus.

### dsIVp53-2 injection impairs virion exit from calyx epithelial cells

**Fig 6:**
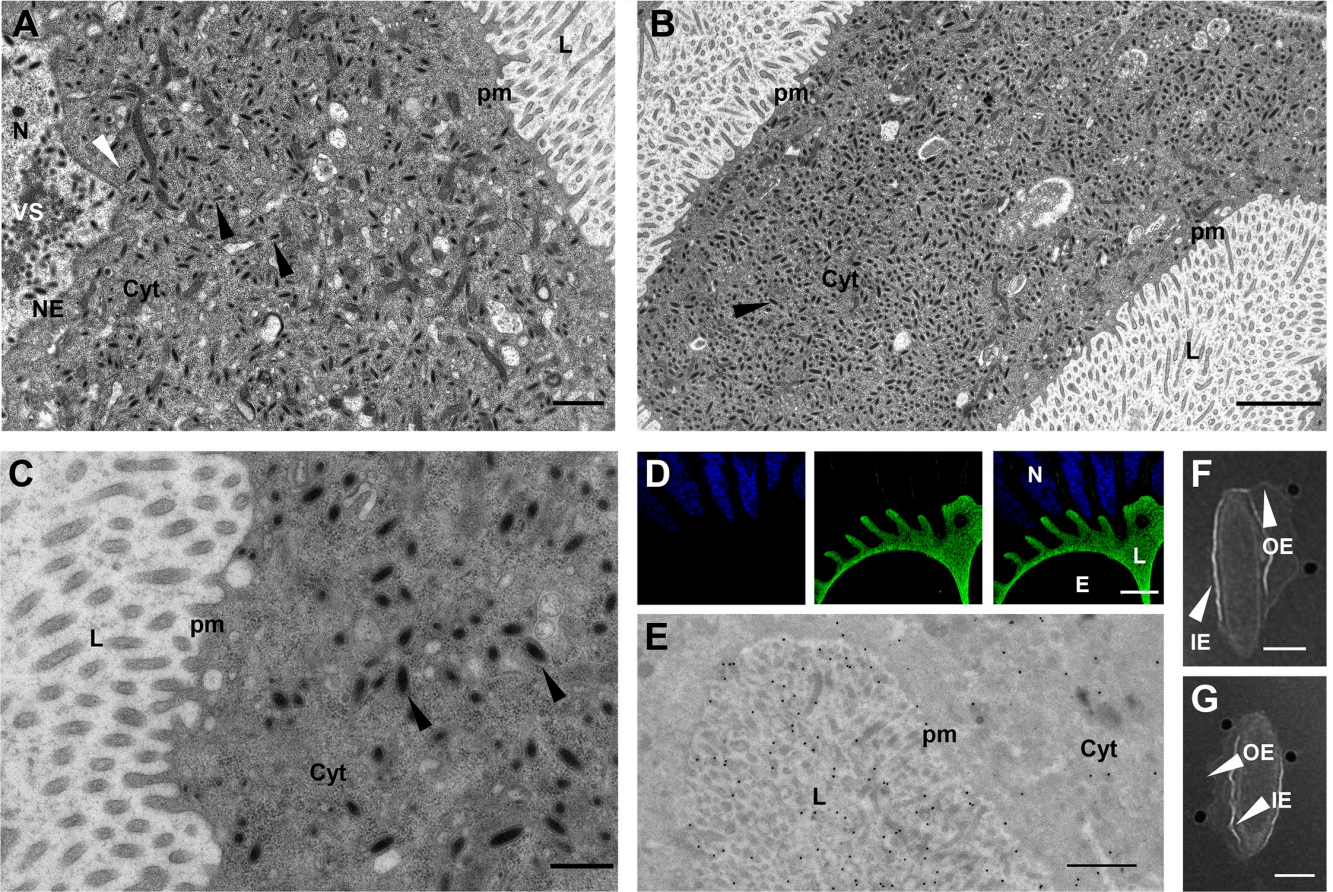
Knockdown of *IVp53-2* impairs subvirion exit from the calyx. (A) General view of dsIVp53-2 treated *H*. *didymator* female showing subvirions formed at the virogenic stroma (VS) in the nucleus (N); after budding through the nuclear envelope (NE), they accumulate in the cytoplasm (Cyt). White arrowhead indicates a subvirion still surrounded by the NE and black arrowheads single-enveloped virions. Note absence of virions budding at the plasma membrane (pm) and inside the calyx lumen (L). (B) View of another calyx cell showing a large amount of subvirions (arrowhead) in the cytoplasm. (C) View of a calyx cell illustrating absence of budding from the cytoplasm into the calyx lumen in dsIVp53-2 treated females. Two subvirions are indicated by arrowheads. (D) Confocal laser scanning image of calyx cells from dsIVp12-1 injected females after immunolabeling using an anti-IVp53-2 antibody (green labeling). Calyx cell nuclei are labeled in blue (DAPI staining). In dsIVp12-1 samples, only the envelopes formed in the nuclei are transferred to the calyx lumen; nucleocapsids are retained in the nucleus. Labeling shows association of IVp53-1 with the envelopes released in the calyx lumen. (E) Immunogold labeling using anti-IVp53-2 antibody showing association of IVp53-2 with viral envelopes in the calyx lumen of dsIVp12-1 treated females. (F and G) Immunogold labeling of viral particles purified from untreated females showing preferential association of IVp53-2 with the outer envelope of the mature virions. E, egg; IE, inner envelope; OE, outer envelope. Scale bars: A: 1 μm; B: 2 μm; C: 500 nm; D: 20μm; E: 1 μm; F, G: 100 nm.

Knockdown of *IVp53-2* resulted in the absence of virus particles within the oviduct of dsRNA-injected females. Earlier stages of viral morphogenesis were apparently not affected. Indeed, subvirions were observed inside the nuclei and the cytoplasm of calyx cells (Fig 6A). However, in dsRNA-injected females, subvirions accumulated to large numbers in the cytoplasm (Fig 6B) but no budding through the apical plasma membrane could be observed (Fig 6C); accordingly, no virions were released into the lumen of the calyx. These observations suggest that IVp53-2, a protein 334 amino acids long that contains a predicted transmembrane domain in its C-terminal region, might be an envelope protein involved in virion budding.

The specific location of IVp53-2 was studied by immuno-labeling using dsIVp12-1 injected females and a specific antibody. Labeling was mainly observed in the calyx lumen (Fig 6D), which contains only virion envelopes in dsIVp12-1 injected wasps. To determine if IVp53-2 colocalizes with the inner (acquired in the nucleus) or the outer (acquired by budding through the plasma membrane) envelope, immunogold labeling was performed on the same dsIVp12-1 treated samples. Labeling was mainly observed in the calyx lumen (Fig 6E) and not in the nuclei, which suggests that IVp53-2 is associated with the outer and not the inner viral envelopes. This was supported by analysis of mature virions collected from untreated wasps; signal was indeed preferentially localized on the virion outer envelopes (Fig 6F and 6G). Based on these observations, IVp53-2 is most probably a protein associated with the virion outer envelope, and necessary for virion budding through the apical plasma membrane.

## Discussion

**Fig 7:**
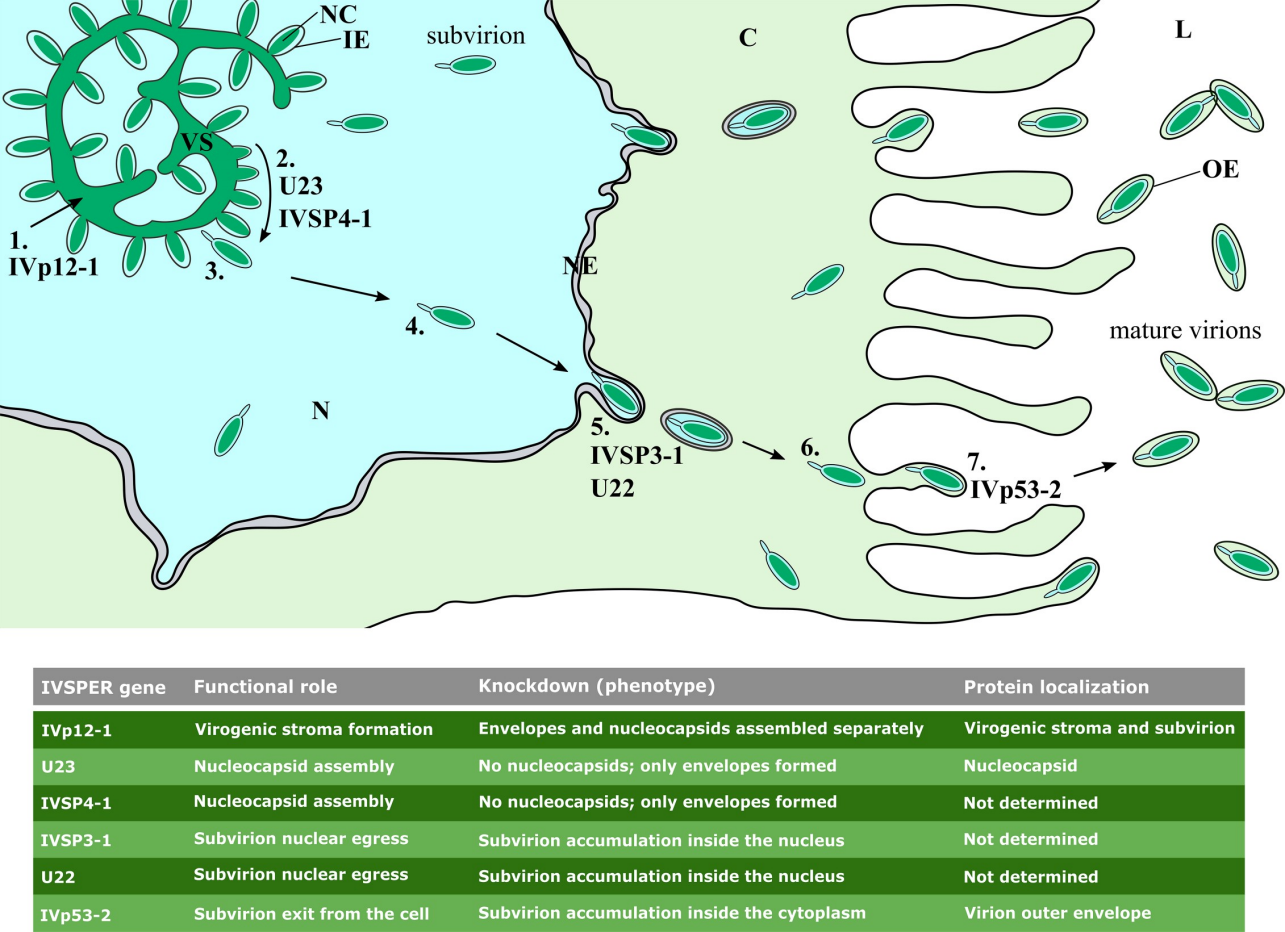
Involvement of IVSPERs genes in ichnovirus morphogenesis. Schematic representation of the different stages in IV morphogenesis. For each stage, the IVSPER genes potentially involved are indicated. **1.** Virogenic stroma formation. **2.** Nuclear subvirion assembly. **3 & 4.** Subvirion dissociation from the virogenic stroma and trafficking through the nucleoplasm. **5.** Subvirion budding through the nuclear envelope. **6.** Subvirion trafficking in the cytoplasm towards the lumen side of the cell. **7.** Virion budding through the plasma membrane. Mature virions with 2 envelopes are stored in the calyx lumen. C, cytoplasm; L, lumen; IE, virion inner envelope; N, nucleus; NC, nucleocapsid; NE, nuclear envelope; OE, virion outer envelope; VS, virogenic stroma. A table synthesizing the findings of the work is presented at the bottom.

Polydnaviruses are unique virus-derived particles produced by parasitic wasps that rely on them to successfully develop in their insect hosts; however, a viral origin for these particles has only recently become clear. The two recognized PDV taxa, the BVs and IVs, are today known to have originated following the acquisition of two different viral genomes by ancestral parasitic Hymenoptera [24]. The BVs, so named because of their association with braconid microgastrine parasitoids, derive from a captured nudivirus genome [17], and are now carried by thousands of wasp species. The IVs, carried by large numbers of ichneumonid parasitoids, originated following the integration of an extinct or currently unknown viral genome [18]. While the function of BV genes descending from the nudiviral ancestor can be inferred by virtue of their homology to core nudivirus genes, the function of IVSPER-located IV genes has been unclear, due to the absence of homologous sequences in current DNA databases. In the present study, we have used RNA interference combined with TEM in an attempt to identify putative IVSPER functions. We initially searched for genes that were upregulated in stage 3 pupae, when the first particles are observed in calyx cell nuclei. In several viruses, including baculoviruses [25] and nudivirus-derived BVs [17,26], the temporal expression pattern of viral genes matches well with their function in the viral cycle: “early” transcribed genes mainly encode proteins involved in the replication of the viral genome and/or in the regulation of viral gene expression, whereas “late” genes mostly encode structural proteins (capsids and/or envelope proteins). We thus selected six IVSPER genes meeting the criterion of late expression and which in addition were predicted to encode virion structural proteins [18]. To specifically inhibit gene expression in *H*. *didymator*, we injected young pupae with gene-specific dsRNAs. In so doing, we have been able to assign putative functions to IVSPER genes. We identified functions related to subvirion and nucleocapsid assembly, exit of subvirions from calyx cell nuclei, and release of virions at the apical cell surface (summarized in Fig 7).

In *H*. *didymator*, subvirion formation involves co-assembly of nucleocapsid and the virion inner membrane on the surface of a virogenic stroma, a structure so named by analogy with baculovirus viral factories [27,28]. Non-enveloped nucleocapsids were never observed in calyx cell nuclei, even at the first steps of virion formation in stage 3 pupae, and therefore do not serve as precursors in the normal virion assembly process. This process appears to be unique in virology. In baculoviruses, a well-studied example of a nuclear-replicating virus that acquires an envelope within the nucleus, nucleocapsids are formed prior to envelopment, which does not occur in association with a virogenic stroma [29]. The process by which subvirions are assembled in *H*. *didymator* suggests that, in this model, all components required (i.e., DNA, proteins, and inner virion envelopes) are located within the virogenic stroma. Our study suggests that IV nucleocapsid envelopment requires IVSPER gene *IVp12-1*. IVp12-1 belongs to a gene family composed of 3 known genes [18] encoding proteins with similarities with one of the first ichnovirus structural protein to be described, the *Campoletis sonorensis* p12 [30]. If expression of *IVp12-1* is inhibited, both nucleocapsids and virion inner membranes are made, but do not interact with each other. Membranes are formed in association with virogenic stroma similar or identical to that observed in control wasps. They then traffic in exactly the same way as normal subvirions, both toward and through the nuclear envelope, finally exiting through the plasma membrane, suggesting that these membranes in fact represent virion inner envelopes. In parallel, non-enveloped nucleocapsids are observed gathered in large inclusions in subnuclear regions other than the virogenic stroma. It is difficult to speculate how separation between nucleocapsids and membranes could occur in dsIVp12-1 injected wasps. In untreated *H*. *didymator* wasps, IVp12-1 is associated with the virogenic stroma and the subvirion. One possibility is that IVp12-1 is normally imported into the virogenic stroma, where it is necessary for proper recruitment of nucleocapsid precursors. IVp12-1 could function as some baculovirus proteins, namely IE1, LEF-3 and P143, which are needed to establish a nuclear domain exhibiting a recruiting ability for other virogenic stroma components [31]. Alternatively, perhaps temporal separation of nucleocapsid and membrane assembly is occurring in dsIVp12-1 injected wasps, such that nucleocapsids are made earlier, and then group together. Our micrographs show no apparent association between nucleocapsids and the virogenic stroma-like structure, but this might possibly be observed at earlier time points.

The origin of the subvirion envelope remains puzzling. Our micrographs did not reveal any obvious physical connection to pre-existing cellular membranes. Nevertheless, we cannot exclude that envelopes may originate from cellular membranes. In the case of enveloped baculovirus nucleocapsids, membranes are thought to derive from microvesicles originating from the nuclear envelope [29,32]. Alternatively, IV envelopes may assemble *de novo* in the nucleus as previously proposed [21], suggesting in turn that open-ended membrane precursors must exist, which many think unlikely. That said, existence of such membranes has been also suggested for nucleocytoplasmic large DNA viruses such as vaccinia virus or mimiviruses [33,34]. In addition, evidence for *de novo* envelope assembly has been reported for at least one nuclear polyhedrosis virus [35]. In the future, isolation of lipid membranes from *H*. *didymator* virus derived particles produced in dsRNA injected wasps may allow comparison of their biochemical composition with wasp cellular membranes, thereby facilitating an investigation into the origin of the particle envelopes.

Complete nucleocapsid assembly is only observed when the expression of IVSPER genes *U23* and *IVSP4-*1 is not impaired. When their expression was inhibited, only empty envelopes were produced. Our work established that following knockdown of either of the two genes, circular DNA species were available within calyx cell nuclei, but were not encapsidated. Many viruses assemble their capsids around their genomes while others, such as baculoviruses and herpesviruses, form first a capsid shell and then package their genome thanks to viral packaging motor proteins [36,37]. In *H*. *didymator*, empty capsids have never been observed in association with virion morphogenesis, suggesting that DNA packaging probably occurs very rapidly or concomitant with capsid assembly. Whether these two proteins, and particularly U23 which co-localizes with nucleocapsids, are truly structural nucleocapsid proteins, or are necessary to initiate DNA packaging and nucleocapsid assembly, remains to be determined.

The exit of *H*. *didymator* subvirions from the nucleus requires IVSPER genes *U22* and *IVSP3-1*. When expression of these genes was inhibited by cognate dsRNAs, subvirions accumulated within nuclei in large numbers. Lack of only one of the two proteins clearly impaired nuclear egress, although some, but not many, particles reaching the edge of the nucleus were apparently able to traverse it and to reach the oviduct lumen. Absence of U22 or IVSP3-1 seems thus to inhibit the ability of the neoformed subvirions to reach the nucleus periphery and/or to compromise their ability to bud through the nuclear envelope. Our results show that envelopes lacking nucleocapsids are able to traffic as normal subvirions, which suggests that nuclear egression depends on factors associated with the envelope rather than with the nucleocapsids. So IVSP3-1 and U22 could be proteins which are associated with the envelope or interact with envelope proteins and which mediate intranuclear transport of subvirions and/or facilitate virion budding. Whether or not virus-derived particles in *H*. *didymator* use actin-based motility as do the baculoviruses [37–39], remains to be investigated.

The final step in particle morphogenesis involves budding of the subvirion through the apical plasma membrane. This results in the acquisition of a second (outer) envelope, and the release of virions into the lumen of the oviduct. Expression of IVSPER gene *IVp53-2* is clearly required for this event, as in its absence, mature extracellular virions are not produced. The *p53* gene was originally identified in *Campoletis sonorensis* and described as encoding a structural polypeptide associated to the inner envelope [40]. In *H*. *didymator*, two genes similar to *C*. *sonorensis* p53 were found, named *IVp53-1* and *IVp53-2*, both containing a predicted transmembrane domain in their C-terminal region and cytoplasmic tail domains [18]. Knockdown of *IVp53-1* has been performed but did not lead to a totally clear phenotype; however, we noted an accumulation of subvirions within the nuclei in some samples, suggesting a possible involvement of IVp53-1 in budding trough the nuclear envelope. Although this remains to be determined, IVp53-1 could be associated with the particle inner envelope, as described for *C*. *sonorensis* p53. Regarding IVp53-2, immunohistochemical and immunogold staining both indicate an association of the protein with the virion outer envelope, suggesting in turn that this protein becomes associated with the apical plasma membrane, and promotes budding through it. Proteins addressed to the plasma membrane of infected cells and essential for virus budding have been reported in several free-living viruses, such as the baculoviruses which rely for that on the envelope glycoprotein GP64, and particularly its cytoplasmic tail domain [41,42]. Further work is now needed to identify/characterize the cellular membrane budding platforms of virus-derived particles to better understand the mechanisms by which budding takes place and what is the role of IVp53-2 in this process.

To the best of our knowledge, the data presented in this paper represent the first functional analysis of virus-derived particle production in an ichneumonid wasp. Prior to this study, we knew only that the ichnovirus lineage derives from the capture of an unknown viral genome [18,20]. While the main steps of particle production had been studied in the past using TEM, nothing was known about how the genes involved, comprising the so-called IVSPER genes, functioned. Here we have shown that RNA interference is an effective and powerful tool for investigating the functions of these presumably ancient genes. In so doing, we show that IVSPER genes are required for particle morphogenesis and trafficking (Fig 7), and that their functions are those expected of a typical virus.

The production of virus-derived particles in ichneumonid wasps is in many ways unique, as is also true for the BVs. In both cases, an ancestral lineage of parasitic wasps has captured an entire viral genome, and repurposed it as a gene delivery vehicle, packaging insect virulence genes rather than viral genes. While prokaryotic gene transfer agents share some features in common with PDVs, there is no absolute requirement for them to be carried by their bacterial hosts. On the other hand, successful parasitism by PDV-bearing wasps is absolutely dependent upon the expression of virulence genes delivered by PDVs into parasitized hosts. That this scenario involves the domestication of two entirely different viruses, an idea supported by the current study, constitutes an astonishing example of convergent evolution subsequent to the integration of these viral genomes into eukaryotic chromosomal DNA.

## Materials and methods

### Insects

Rearing of *Hyposoter didymator* wasps was conducted as previously described [43]. Female pupae extracted from cocoons were classified into different stages according to their pigmentation pattern: stage 1, for hyaline pupae (~3 day-old pupae); stage 2, for pupae with pigmented thorax (~4 day-old pupae); stage 3, for pupae with pigmented thorax and abdomen (~5 day-old pupae); stage 4, for adults prior to emergence (~6 day-old pupae).

### Total RNA extraction

For transcriptome analyses, total RNAs were extracted from ovaries (ovarioles removed) dissected from pupae at different stages using the Qiagen RNeasy extraction kit according to manufacturer’s protocol. To control for gene silencing, total RNAs were extracted from individual adult wasp abdomens (2 to 4 days-old) first using Trizol reagent (Ambion) and then the NucleoSpin RNA kit (Macherey-Nagel) according to the manufacturer’s recommendations. Following extraction, total RNAs were systematically submitted to DNase treatment with the TURBO DNA-free Kit (Life Technologies).

### Reverse-transcriptase quantitative real-time PCR (RT-qPCR)

For RT-qPCRs, 400 ng of purified RNA were reverse-transcripted with the SuperScript III Reverse Transcriptase kit (Life Technologies) and oligo(dT)15 primer (Promega). The mRNA transcripts level of selected IVSPER genes was measured by qRT-PCR using a LightCycler 480 System (Roche) and SYBR Green I Master Mix (Roche) and was normalized relative to a housekeeping wasp gene (elongation factor 1 (*ELF-1*)). Each sample was evaluated in triplicate. The total reaction volume per well was 3μl including 0.5 μM of each primer and cDNA corresponding to 0.88 ng of total RNA and the program for amplification was 95°C for 10 min, followed by 45 cycles of 95°C for 10 s, 58°C for 10 s, and 72°C for 10 s. Primers used are listed in S5 Table.

### RNA-seq analysis

Total RNAs were extracted from pools of 40 to 50 ovaries dissected from females at pupal stages 1, 2 and 3. Three replicates were collected for each pupal stage. DNase-treated RNAs were then sent to the Genewiz company (https://www.genewiz.com) for sequencing (1x50bp SR, HiSeq2500). A total of ~2.E+07 reads (~1000 Mbase) were obtained per sample. Reads were mapped on the wasp genome using STAR aligner version 2.5.2a [44] with the default parameters except the following parameters: outFilterMultimapNmax = 5, outFilterMismatchNmax = 3, alignIntronMin = 10, alignIntronMax = 50000 and alignMatesGapMax = 50000. Read counting was performed with the tool FeatureCounts version 1.5.0-p3 [45] with the default parameters except the following parameters: -g Parent, -M—fraction, and analyzed with package EdgeR [46]. For selected genes, levels of mRNA were validated by RT-qPCR using total RNAs extracted from pools of 15 to 25 ovaries collected from pupae at 4 different stages (1, 2, 3 and 4).

### RNA interference (RNAi)

The gene-specific double-stranded RNA (dsRNA) used for RNAi experiments were prepared with the T7 RiboMAX Express RNAi System (Promega) according to the manufacturer’s instructions. Briefly, for each gene, a 600–750 bp fragment was cloned into the double T7 vector L4440 (a gift from Andrew Fire (Addgene plasmid # 1654)). Then, a transcription *in vitro* template DNA was PCR amplified with T7 primer and used to synthetized sense and antisense RNA strands with T7 RNA polymerase at 37°C during 5 h. The primers used to produce dsRNA are indicated in S5 Table. After annealing and DNase treatment with the TURBO DNA-free Kit (Life Technologies), purified dsRNA were resuspended in nuclease-free water, quantified using a NanoDrop ND-1000 Spectrophotometer (Thermo Scientific) and examined by agarose gel electrophoresis to ensure their integrity. Injections were performed in less than one-day old female pupae using a microinjector Fentojet Express (Eppendorf) and a micromanipulator (Narishige). Approximately 0.3–0.6 μl of 2–4 μg/μl dsRNA was injected into each individual. Control wasps were injected with a non-specific dsRNA homologous to the green fluorescent protein (GFP) gene. Treated pupae were kept in an incubator until adult emergence, which occurred approximately 5 days after injection. All the pupae that survived emerged as adults with apparently normal egg loads.

### Transmission electron microscopy

Ovaries were dissected from adult wasps between 2 and 3 days after emergence, and treated as described in [47]. At least three females (taken at different microinjection dates) were observed for each tested dsRNA to ensure consistency of the observed phenotype. For TEM observations, calyces were fixed 2 hours in a solution of 2% glutaraldehyde in PBS and post fixed in 2% tetroxide osmium in the same buffer for 1 h. Tissues were then bulk-stained for 2 hours in 5% aqueous uranyl acetate, dehydrated in ethanol and embedded in EM812 resin (EMS). Ultrathin sections were double stained in Uranyless (DeltaMicroscopy) and lead citrate and examined under a Jeol 1200 EXII electron microscope at 100 kV (MEA Platform, University of Montpellier). Images were taken with an EMSIS Olympus Quemesa 11 Mpixels camera and analyzed with ImageJ software [48].

### Semi-thin sections

Ovaries were dissected out of dsRNA treated female wasps then fixed in 4% PFA, dehydrated in ethanol and then embedded in Unicryl resin (EMS) for semi-thin sections. Sections were stained with 1% toluidine blue in 1% sodium borate, or DAPI (4',6-diamidino-2-phenylindole) at 1μg/ml, for observation by light and fluorescence microscopy, respectively. Five different females, taken at 2 different microinjection dates, were observed for each tested dsRNA to ensure reliability of the observed phenotype. Fluorescence was recorded using a laser scanning confocal microscope (Leica microsystems TCS SPE) on MRI platform (University of Montpellier). Images were taken using the software LAS AF (Leica).

### Western blot analysis and immunofluorescence

Custom polyclonal antibodies from peptides based on U23, IVp12-1 and IVp53-2 amino acid sequences were synthesized by Eurogentec (https://secure.eurogentec.com/life-science.html). Two peptides were selected per genes to obtain U23 rat, IVp53-2 rabbit and IVp12-1 Guinea pig antiserum (sequences in S6 Table). Western-blots were performed to control the antibody specificity (see S3 Fig). Briefly, proteins were extracted from 10 calyces of dsRNA injected or untreated wasps, separated on 10% polyacrylamide gels and electro-transferred to PVDF membranes (ThermoFisher Scientific). Blots were blocked with 5% low fat-milk, 0.05% Tween in TBS and incubated overnight at 4°C with anti-U23, -IVp53-2, -IVp12-1 sera at dilutions of 1:500. Antibody binding was detected with peroxidase-conjugated secondary antibodies (ThermoFisher Scientific) and visualized by chemiluminescence (LI-COR Odyssey FC imaging system). For immunofluorescence, semi-thin sections of calyces were incubated with rat anti-U23, rabbit anti-IVp53-2 or guinea pig anti-IVp12-1 (1:1000) sera in PBS containing 0.1% bovine serum albumin (BSA) and overlaid with Alexa fluor 594 goat, anti-rat IgH (Invitrogen), Alexa fluor 488 donkey, anti-rabbit (Invitrogen) or Alexa fluor 647 goat, anti-guinea pig (Abcam). After each step, semi-thin sections were washed repeatedly with PBS and finally stored at 4°C. Fluorescence was recorded using a laser scanning confocal microscope (Leica microsystems TCS SPE) on MRI platform (University of Montpellier). Images were taken using the software LAS AF (Leica).

### Immunolabeling

For TEM immunogold labeling, calyces were treated as described in [26]. Reaction of the anti-IVp12-1, anti-U23 and anti-IVp53-2 sera were visualized by binding of anti-rat (10nm diameter gold beads), anti-rabbit (20nm diameter gold beads) and anti-guinea pig (10nm diameter gold beads) antibodies (Aurion EM reagents). Ultrathin sections were examined under a Jeol 1200 EXII electron microscope at 100 kV (MEA Platform, University of Montpellier). Images were taken with an EMSIS Olympus Quemesa 11 Mpixels camera and analyzed with Image J software [48].

### PCR-based detection of packaged DNA

HdIV particles were purified from calyces of 2 day-old wasp females as described in [49]. To avoid contamination with non-packaged DNA, particles were submitted to a DNase treatment using the Turbo DNA-free kit (Life Technology/Invitrogen) according to the manufacturer’s protocol. DNA was subsequently extracted from the particles according to standard protocols [49] and used as a template for PCR amplification of the HdIV gene *GlyPro2* (GenBank: AIK25735.1). The IVSPER gene *U23* and the wasp *COI* gene were used to control absence of genomic contamination in the samples (primer sequences are available in S5 Table). The control consisted of DNA extracted from HdIV particles collected from untreated females and not subjected to DNase treatment.

### PCR-based detection of circularized HdIV DNA

Genomic DNA was extracted from isolated ovaries dissected from 48 h old females, using the QuickExtract^T^ Bacterial DNA extraction Kit (Epicentre/TEBU-BIO) according to the manufacturer’s instructions. Genomic DNA extracted from males was used as a negative control for viral DNA circularization. Genomic DNA was used as a template for PCR amplification using primers specific to HdIV segment 29 (Hd29, GenBank: KJ586303.1) and using primer pairs that were designed for amplification of either the integrated sequence (HdBR-RR/HdBR-RL) or the episomal Hd29 sequence (HdBR-LL/HdBR-RR) (primer sequence in S5 Table).

### qPCR data analysis

Data were collected using Light-Cycler 480 software. PCR amplification efficiency (*E*) for each primer pair was determined by linear regression of a dilution series (5x) of cDNA pool. Relative expression using the housekeeping gene *ELF-1* as a reference was calculated using advanced relative quantification (Efficiency method) software provided by Light-Cycler 480 software [50]. For statistical analyses, Levene’s and Shapiro-Wilk tests were used to verify homogeneity of variance and normal distribution of data among groups tested. Differences in gene relative expression between the 4 developmental stages and between dsGFP and dsRNA injected females were tested using two-tailed unpaired t-test for group comparison. When homogeneity of variance was not assumed, Welch-test was used to compare gene relative expression between groups. A p-value < 0.05 was considered significant. All statistical analyses were performed with R [51].

## Supporting information

S1 Fig Validation by RT-qPCR of RNAi knockdown of targeted IVSPER genes. Relative expression of selected IVSPER genes in dsGFP (control) and dsRNA injected females. Transcription levels were analyzed for the 6 genes studied in this work plus *IVp53-1* (NCBI protein_id = ADI40489.1) which shares 83% nucleotide identity with *IVp53-2*. ns = non-significant, *p<0.005, **p<0.01 and ***p<0.001. The y axis was transformed by the square root function for better visualization.
(DOCX)

S2 Fig Analysis of the DNA content of defective particles produced in wasps injected with dsU23 and dsIVSP4-1 RNAs. **A. Detection of viral DNA within purified particles (PCR).** PCR amplification results from viral particles purified from dsRNA injected wasps (G: injected with dsGFP; U: injected with dsU23; I: injected with dsIVSP4-1). Controls (C) corresponded to HdIV DNA extracted from particles from non-treated females. Primers were specific either to the packaged HdIV gene *GlyPro2* (encoded by segment Hd2a, GenBank: KJ586332; control of presence of packaged DNA in the purified particles) and to the cytochrome oxidase I (COI) wasp gene (control of gDNA contamination). Amplification of packaged DNA (GlyPro2 lanes) was observed only in the dsGFP samples (G1 and G2). **B. DAPI staining.** DNA labeling of calyces dissected from dsGFP, dsU23 and dsIVSP4-1 injected *H*. *didymator* females. DNA was stained either with toluidine blue (left panels) or with DAPI (right panels). DNA labeling was observed in the calyx lumen (arrows, right panels) only in dsGFP treated females, indicating absence of packaged HdIV DNA following silencing of *U23* or *IVSP4-1*. C = calyx cells, E = egg, L = calyx lumen. DIC = differential interference contrast. **C. Detection of circular viral DNA in the calyx cells.** PCR Amplification of circular and chromosomal forms of HdIV segment 29 (GenBank: KJ586303.1) in females injected with either dsGFP, dsIVSP4-1 or dsU23. The diagram indicates the position and orientation of the PCR primers used in the experiment. PCR was performed using genomic DNA extracted from wasp abdomens and primers specific for the chromosomal (LL+LR, RR+RL) or circular form (RR+LL, Seg29). The samples tested were either males (negative control; no episomal form expected in these samples; n = 4) or 2 day old females treated with dsGFP (n = 6), dsU23 (n = 7) or dsIVSP4-1 (n = 10); only 2 samples are shown for each treatment in the figure.
(DOCX)

S3 Fig Validation of antibody specificity by Western-blotting. Antibodies directed against IVp12-1, U23 and IVp53-2, respectively were tested on proteins extracted from calyces dissected from either untreated *H*. *didymator* females (Positive control; right lanes) or of dsRNA injected females (left lanes). Red rectangles indicate antibody labeling on control protein extracts and absent or reduced labeling in dsRNA samples. Note that molecular weight observed for labeled bands is higher than expected (8 kDa for IVp12-1, 47 kDa for U23 and 36 kDa for IVp53-2), possibly because of post-transductional modification.
(DOCX)

S1 Table Expression of IVSPER genes (RPKM) in *H. didymator* calyces at 3 developmental pupal stages and differential expression between pupal stages. Stage 1: hyaline pupa; stage 2: pupa with pigmented thorax; stage 3: pupa with pigmented abdomen. Statistical analyses were performed using EdgeR package. LogFC = Log2 Fold Change, FDR = False Discovery Rate, ns = non-significant, *p<0.05. U1 data missing.
(XLSX)

S2 Table Relative expression of 6 IVSPER genes in *H. didymator* calyces at 4 pupal developmental stages. Total RNA was extracted from calyces of wasp female pupae at Stage 1: hyaline pupa; stage 2: pupa with pigmented thorax; stage 3: pupa with pigmented abdomen; stage 4: close to adult emergence. The relative gene expression was calculated for each target gene relative to the reference gene ELF-1, using the Advanced Analysis method provided in the Light Cycler 480 system, which considers the PCR efficiencies of the target genes (see below) and the reference gene (ELF-1 efficiency = 1,98). Indicated are the qPCR cycle threshold (Ct) for target and ELF-1 genes and the relative expression of each target gene. N = 3 biological replicas per stages (R1, R2, R3). The "mean Ct" corresponds to the mean value of the 3 technical replicas. Statistical analyses were performed using two-tailed unpaired student t-test statistics. A Levene's test was performed to assess the equality of variances. n.s = non-significant, *p<0.05, **p<0.01 and ***p<0.001.
(XLSX)

S3 Table Comparative expression of IVSPER genes in dsGFP and dsRNA injected females. qPCR cycle threshold (Ct) and relative expression to ELF-1 results were obtained using advanced relative quantification (Efficiency method) provided by Light Cycler 480 software. For each treatment (*i*.*e*. injection of dsRNA targeting a given IVSPER gene versus dsGFP) are indicated the number of biological replicas (C for controls and T for treatments) and the targeted gene. The "mean Ct" corresponds to the mean value of the 3 technical replicas.
(XLSX)

S4 Table Comparative analysis of IVSPER genes expression in dsGFP and dsRNA injected females. Two-tailed unpaired t-test or Welch test statistics. ns = non-significant, *p<0.005, **p<0.01 and ***p<0.001.
(XLSX)

S5 Table List of primers used in the different experiments. (XLSX)

S6 Table Peptides used to generate antibodies directed against IVp12-1, IVp53-2 and U23. (XLSX)
