# Supplementary material for: RNA interference identifies domesticated viral genes involved in assembly and trafficking of virus-derived particles in ichneumonid wasps
Source: PLoS Pathog. 2019 Dec 13;15(12):e1008210. doi: 10.1371/journal.ppat.1008210 (PMC6957214; doi:10.1371/journal.ppat.1008210)

**S1 Fig. Validation by RT-qPCR of RNAi knockdown of targeted IVSPER genes.** Relative expression of selected IVSPER genes in ds-*GFP* (control) and ds-*RNA* injected females; expression given relative to the housekeeping gene ELF-1. For each validation, transcription levels were systematically verified for the 6 genes studied in this work (as well as for *IVp53-1* (NCBI protein_id= ADI40489.1) which shares 83% nucleotide identity with *IVp53-2*). ns= non-significant, *p<0.005, **p<0.01 and ***p<0.001. Axis y was transformed by the square root function for a better data visualization.


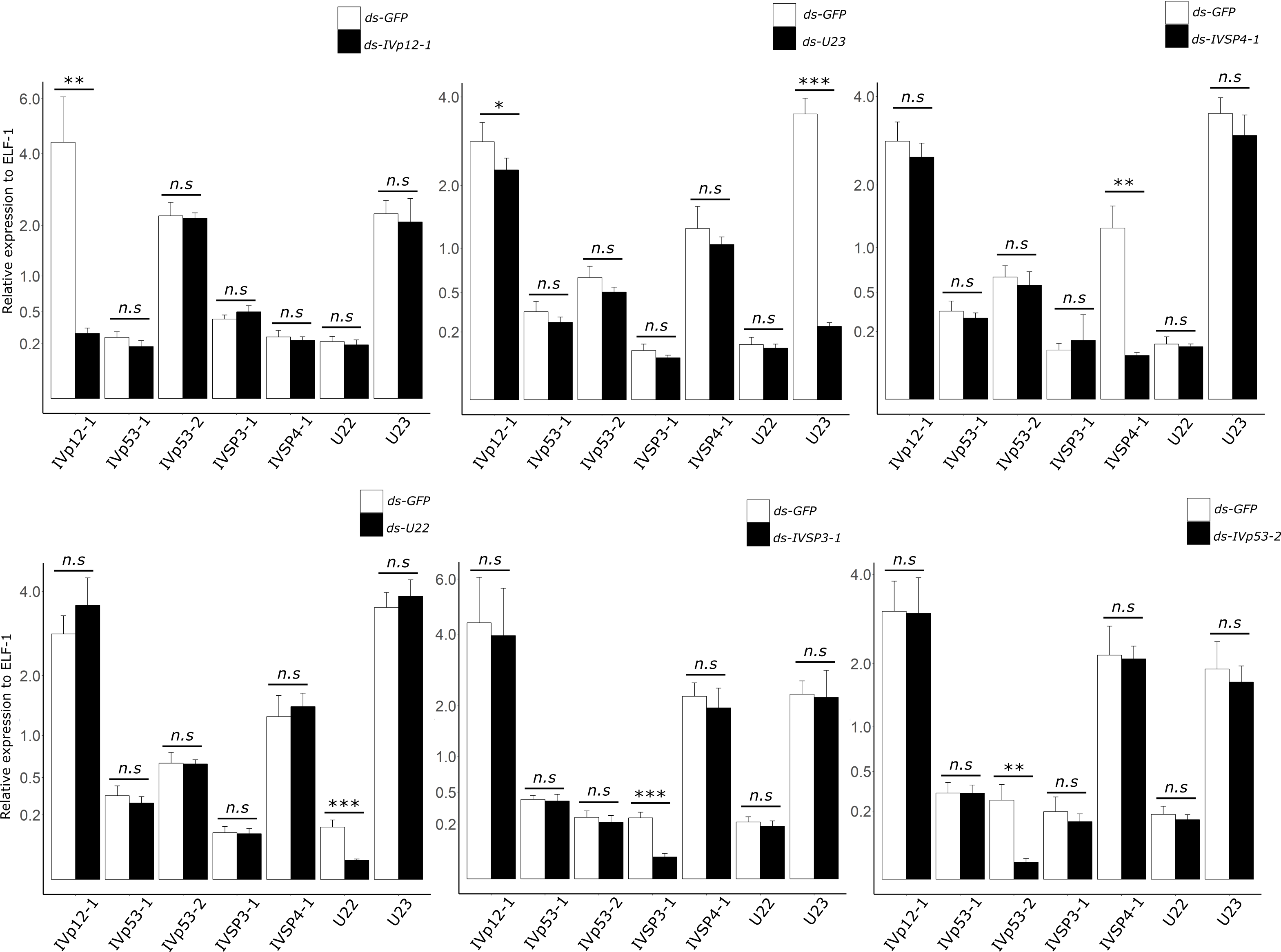

Supplement: S1 Fig — Relative expression of selected IVSPER genes in dsGFP (control) and dsRNA injected females. Transcription levels were analyzed for the 6 genes studied in this work plus IVp53-1 (NCBI protein_id = ADI40489.1) which shares 83% nucleotide identity with IVp53-2. ns = non-significant, *p<0.005, **p<0.01 and ***p<0.001. The y axis was transformed by the square root function for better visualization. (DOCX) [file ppat.1008210.s001.docx]
