# Supplementary material for: RNA interference identifies domesticated viral genes involved in assembly and trafficking of virus-derived particles in ichneumonid wasps
Source: PLoS Pathog. 2019 Dec 13;15(12):e1008210. doi: 10.1371/journal.ppat.1008210 (PMC6957214; doi:10.1371/journal.ppat.1008210)

**S2 Fig. Analysis of the DNA content of defective particles produced in wasps injected with dsU23 and dsIVSP4-1 RNAs.**


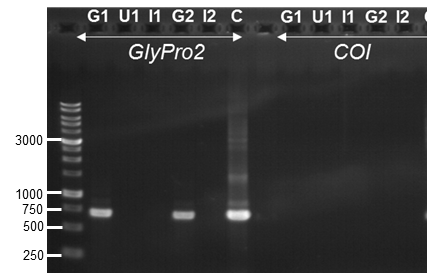
**A. Detection of viral DNA within purified particles (PCR).** PCR amplification results from viral particles purified from ds-RNA injected wasps (G: injected with dsGFP; U: injected with dsU23; I: injected with dsIVSP4-1). Controls (C) corresponded to HdIV DNA extracted from particles from non-treated females. Primers were specific either to the packaged HdIV gene *GlyPro2* (encoded by segment Hd2a, GenBank: KJ586332) (control of presence of packaged DNA in the purified particles; expected size of PCR product 709 bp) and to the wasp gene *COI* (control of gDNA contamination; expected size of PCR product 709 bp). Amplification of packaged DNA (GlyPro2 lanes) was observed only in the dsGFP samples (G1 and G2). PM, Nippon Genetics 1kb (Dutscher).

**B. DAPI staining.** DNA labeling of calyces dissected from dsGFP, dsU23 and dsIVSP4-1 injected *H. didymator* females. DNA was stained either with Toluidine blue (left panels) or with DAPI (right panels). DNA labeling was observed in the calyx lumen (arrows, right panels) only in dsGFP treated females, indicating absence of packaged HdIV DNA following silencing of *U23* or *IVSP4-1*. C = calyx cells, E = egg, L = calyx lumen. DIC= differential interference contrast.


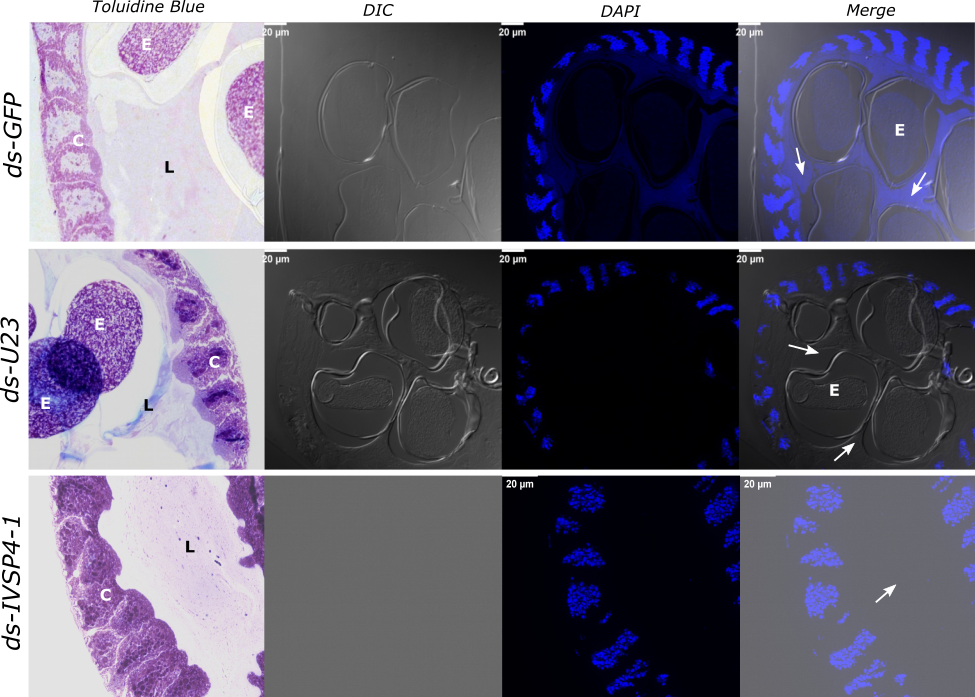


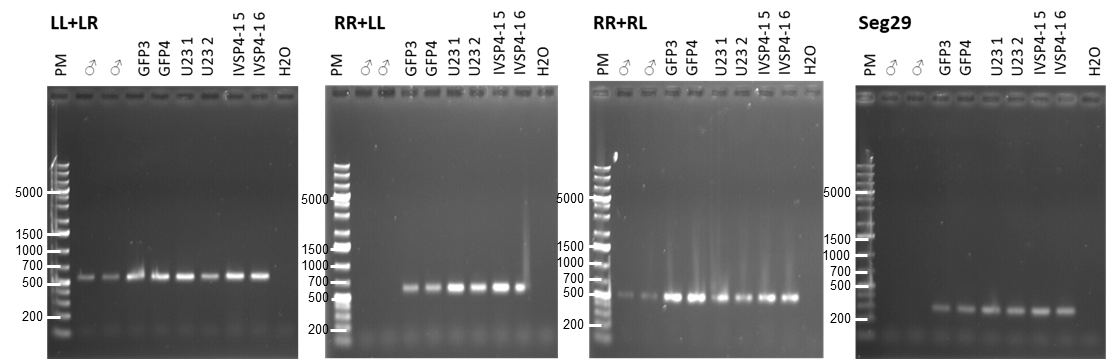
**C. Detection of circular viral DNA in the calyx cells.** PCR Amplificationof the circular and integrated forms of the HdIV segment 29 (GenBank: KJ586303.1) in females injected with either dsGFP, dsIVSP4-1 or dsU23. The diagram indicates the position and orientation of the PCR primers used in the experiment. PCR were performed using genomic DNA extracted from wasp abdomens and primers specific of the integrated (LL+LR, RR+RL) or circular form (RR+LL, Seg29). The samples tested were either males (negative control; no episomal form expected in these samples; n=4) or 2 days old females treated with dsGFP (n=6), dsU23 (n=7) or dsIVSP4-1 (n=10); only 2 samples are shown for each treatment in the figure. Expected size for amplification products: LL+LR, 550 bp; RR+LL, 550 bp; RR+RL, 529 pb; Seg29, 282 bp. PM, Euromedex 1kb DNA Ladder plus.


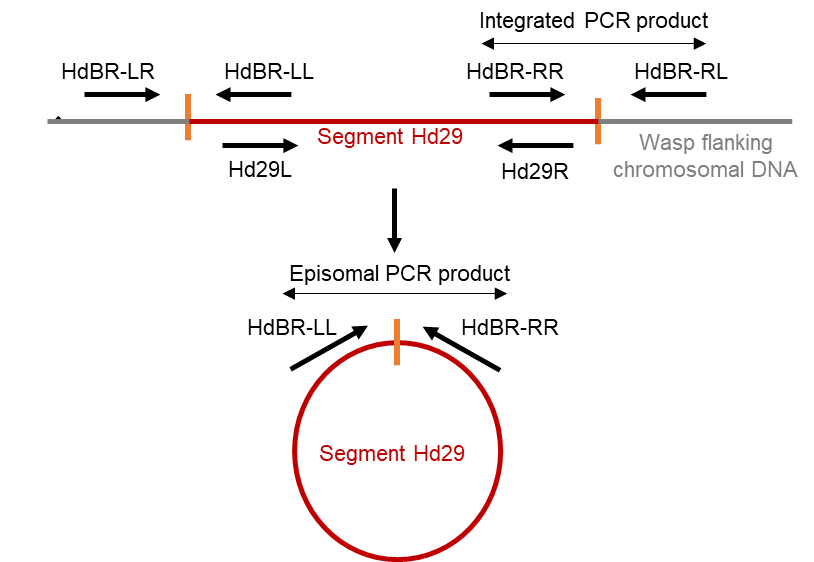

Supplement: S2 Fig — A. Detection of viral DNA within purified particles (PCR). PCR amplification results from viral particles purified from dsRNA injected wasps (G: injected with dsGFP; U: injected with dsU23; I: injected with dsIVSP4-1). Controls (C) corresponded to HdIV DNA extracted from particles from non-treated females. Primers were specific either to the packaged HdIV gene GlyPro2 (encoded by segment Hd2a, GenBank: KJ586332; control of presence of packaged DNA in the purified particles) and to the cytochrome oxidase I (COI) wasp gene (control of gDNA contamination). Amplification of packaged DNA (GlyPro2 lanes) was observed only in the dsGFP samples (G1 and G2). B. DAPI staining. DNA labeling of calyces dissected from dsGFP, dsU23 and dsIVSP4-1 injected H. didymator females. DNA was stained either with toluidine blue (left panels) or with DAPI (right panels). DNA labeling was observed in the calyx lumen (arrows, right panels) only in dsGFP treated females, indicating absence of packaged HdIV DNA following silencing of U23 or IVSP4-1. C = calyx cells, E = egg, L = calyx lumen. DIC = differential interference contrast. C. Detection of circular viral DNA in the calyx cells. PCR Amplification of circular and chromosomal forms of HdIV segment 29 (GenBank: KJ586303.1) in females injected with either dsGFP, dsIVSP4-1 or dsU23. The diagram indicates the position and orientation of the PCR primers used in the experiment. PCR was performed using genomic DNA extracted from wasp abdomens and primers specific for the chromosomal (LL+LR, RR+RL) or circular form (RR+LL, Seg29). The samples tested were either males (negative control; no episomal form expected in these samples; n = 4) or 2 day old females treated with dsGFP (n = 6), dsU23 (n = 7) or dsIVSP4-1 (n = 10); only 2 samples are shown for each treatment in the figure. (DOCX) [file ppat.1008210.s002.docx]
