# Supplementary material for: RNA interference identifies domesticated viral genes involved in assembly and trafficking of virus-derived particles in ichneumonid wasps
Source: PLoS Pathog. 2019 Dec 13;15(12):e1008210. doi: 10.1371/journal.ppat.1008210 (PMC6957214; doi:10.1371/journal.ppat.1008210)

**S3 Fig. Validation by Western-blot of the antibody specificity.** Antibodies directed against IVp12-1, U23 and IVp53-2, respectively were tested on proteins extracted from calyces dissected from either untreated *H. didymator* females (Positive control; left lanes) or of ds-RNA injected females (right lanes). Red rectangles indicate antibody labeling on control protein extracts and absent or reduced labeling in ds-RNA samples. Note that molecular weight observed for labeled bands is higher than expected (8 kDa for IVp12-1, 47 kDa for U23 and 36 kDa for IVp53-2) possibly because of post-traductional modifications.


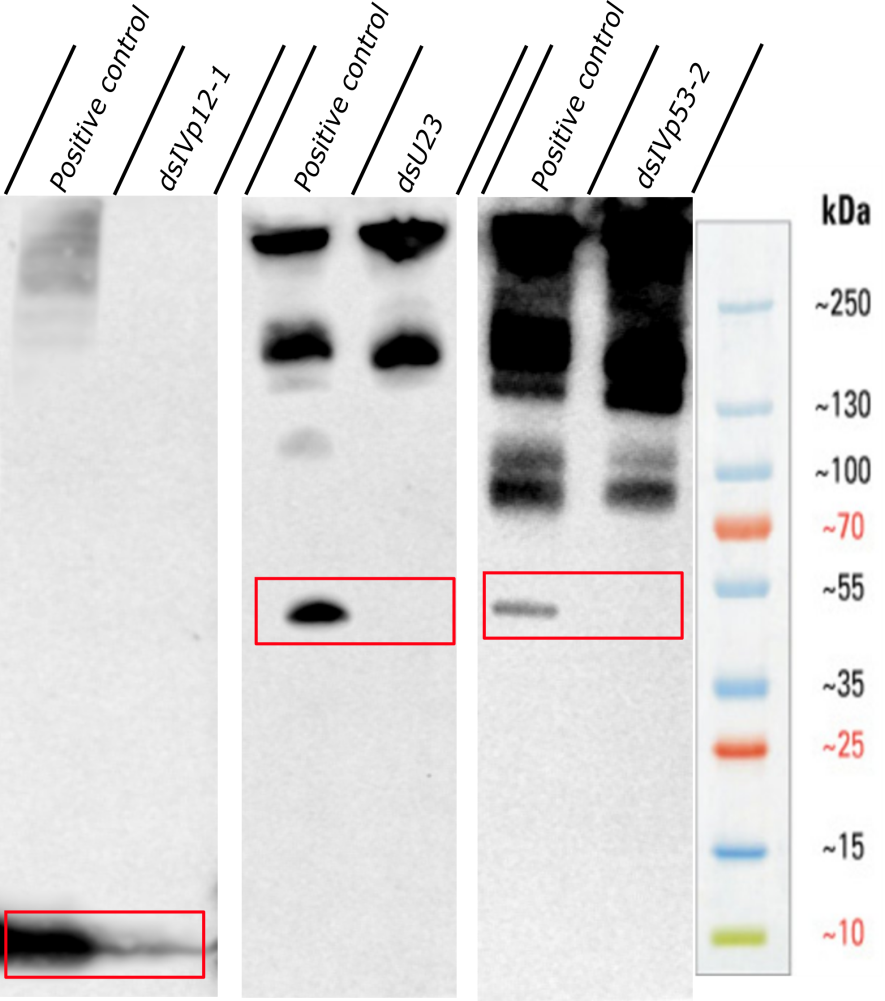

Supplement: S3 Fig — Antibodies directed against IVp12-1, U23 and IVp53-2, respectively were tested on proteins extracted from calyces dissected from either untreated H. didymator females (Positive control; right lanes) or of dsRNA injected females (left lanes). Red rectangles indicate antibody labeling on control protein extracts and absent or reduced labeling in dsRNA samples. Note that molecular weight observed for labeled bands is higher than expected (8 kDa for IVp12-1, 47 kDa for U23 and 36 kDa for IVp53-2), possibly because of post-transductional modification. (DOCX) [file ppat.1008210.s003.docx]
